# Ammonium 4,6-dioxo-2-sulfanylidene-1,3-diazinan-5-ide

**DOI:** 10.1107/S1600536811016722

**Published:** 2011-05-07

**Authors:** Richard Betz, Thomas Gerber

**Affiliations:** a Nelson Mandela Metropolitan University, Summerstrand Campus, Department of Chemistry, University Way, Summerstrand, PO Box 77000, Port Elizabeth 6031, South Africa

## Abstract

In the title salt, NH_4_^+^·C_4_H_3_N_2_O_2_S^−^, the asymmetric unit comprises two half-occupied ammonium positions and a 4,6-dioxo-2-sulfanylidene-1,3-diazinan-5-ide anion. The anion shows *C*_2_ as well as *C*_s_ symmetry and is present in its diketonic tautomeric form. Intra­cyclic angles span a range from 116.64 (9)–124.67 (9)°. Inter­molecular N—H⋯O hydrogen bonds connect the cations and anions to form a three-dimensional network.

## Related literature

For the crystal structures of 2-thio­barbituric acid, its hydrate and several of its tautomeric forms, see: Calas & Martinez (1967 ▶); Chierotti *et al.* (2010 ▶). For graph-set analysis of hydrogen bonds, see: Etter *et al.* (1990 ▶); Bernstein *et al.* (1995 ▶). 
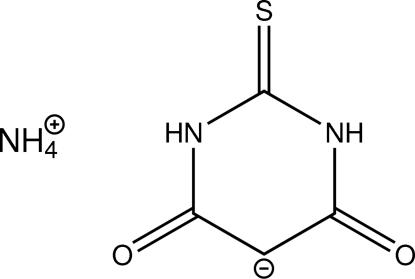


## Experimental

### 

#### Crystal data

NH_4_^+^·C_4_H_3_N_2_O_2_S^−^
*M* *_r_* = 161.19
Monoclinic, 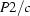

*a* = 11.3308 (4) Å
*b* = 3.9396 (1) Å
*c* = 14.6945 (5) Å
β = 98.644 (1)°
*V* = 648.49 (4) Å^3^
*Z* = 4
Mo *K*α radiation
μ = 0.44 mm^−1^
*T* = 200 K
0.53 × 0.19 × 0.08 mm

#### Data collection

Bruker APEXII CCD diffractometer
Absorption correction: multi-scan (*SADABS*; Bruker, 2008 ▶) *T*_min_ = 0.876, *T*_max_ = 1.000
6071 measured reflections
1604 independent reflections
1476 reflections with *I* > 2σ(*I*)
*R*_int_ = 0.017

#### Refinement

*R*[*F* ^2^ > 2σ(*F* ^2^)] = 0.023
*wR*(*F* ^2^) = 0.067
*S* = 1.10
1604 reflections
116 parameters
H atoms treated by a mixture of independent and constrained refinement
Δρ_max_ = 0.33 e Å^−3^
Δρ_min_ = −0.20 e Å^−3^

### 

Data collection: *APEX2* (Bruker, 2010 ▶); cell refinement: *SAINT* (Bruker, 2010 ▶); data reduction: *SAINT*; program(s) used to solve structure: *SHELXS97* (Sheldrick, 2008 ▶); program(s) used to refine structure: *SHELXL97* (Sheldrick, 2008 ▶); molecular graphics: *ORTEP-3* (Farrugia, 1997 ▶) and *Mercury* (Macrae *et al.*, 2006 ▶); software used to prepare material for publication: *SHELXL97* and *PLATON* (Spek, 2009 ▶).

## Supplementary Material

Crystal structure: contains datablocks I, global. DOI: 10.1107/S1600536811016722/bq2297sup1.cif

Supplementary material file. DOI: 10.1107/S1600536811016722/bq2297Isup2.cdx

Structure factors: contains datablocks I. DOI: 10.1107/S1600536811016722/bq2297Isup3.hkl

Supplementary material file. DOI: 10.1107/S1600536811016722/bq2297Isup4.cml

Additional supplementary materials:  crystallographic information; 3D view; checkCIF report

## Figures and Tables

**Table 1:** Hydrogen-bond geometry (Å, °)

*D*—H⋯*A*	*D*—H	H⋯*A*	*D*⋯*A*	*D*—H⋯*A*
N1—H71⋯O2^i^	0.832 (17)	1.971 (17)	2.8025 (12)	179.0 (16)
N2—H72⋯O1^ii^	0.874 (16)	1.976 (16)	2.8483 (12)	175.7 (15)
N90—H901⋯S1^iii^	0.902 (17)	2.426 (16)	3.3252 (8)	174.8 (14)
N90—H902⋯O2	0.866 (17)	1.927 (17)	2.7929 (12)	177.7 (16)
N91—H911⋯O1^iv^	0.88 (2)	1.90 (2)	2.7622 (13)	166.0 (18)
N91—H912⋯O1^v^	0.87 (2)	2.66 (2)	3.0669 (15)	110.0 (16)
N91—H912⋯S1^vi^	0.87 (2)	2.62 (2)	3.3069 (3)	136.0 (19)

Symmetry codes: (i) 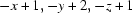; (ii) 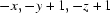; (iii) 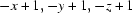; (iv) 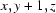; (v) 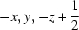; (vi) 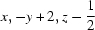.

**Fig. 1.**
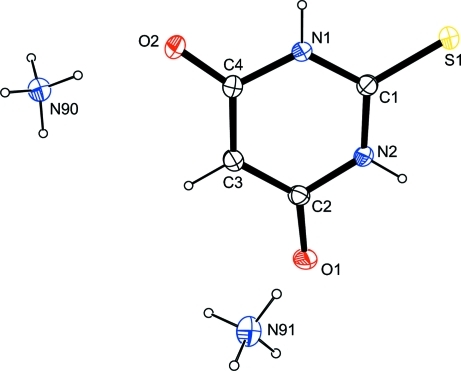
The molecular structure of the title compound, with atom labels and anisotropic displacement ellipsoids (drawn at 50% probability level).

**Fig. 2.**
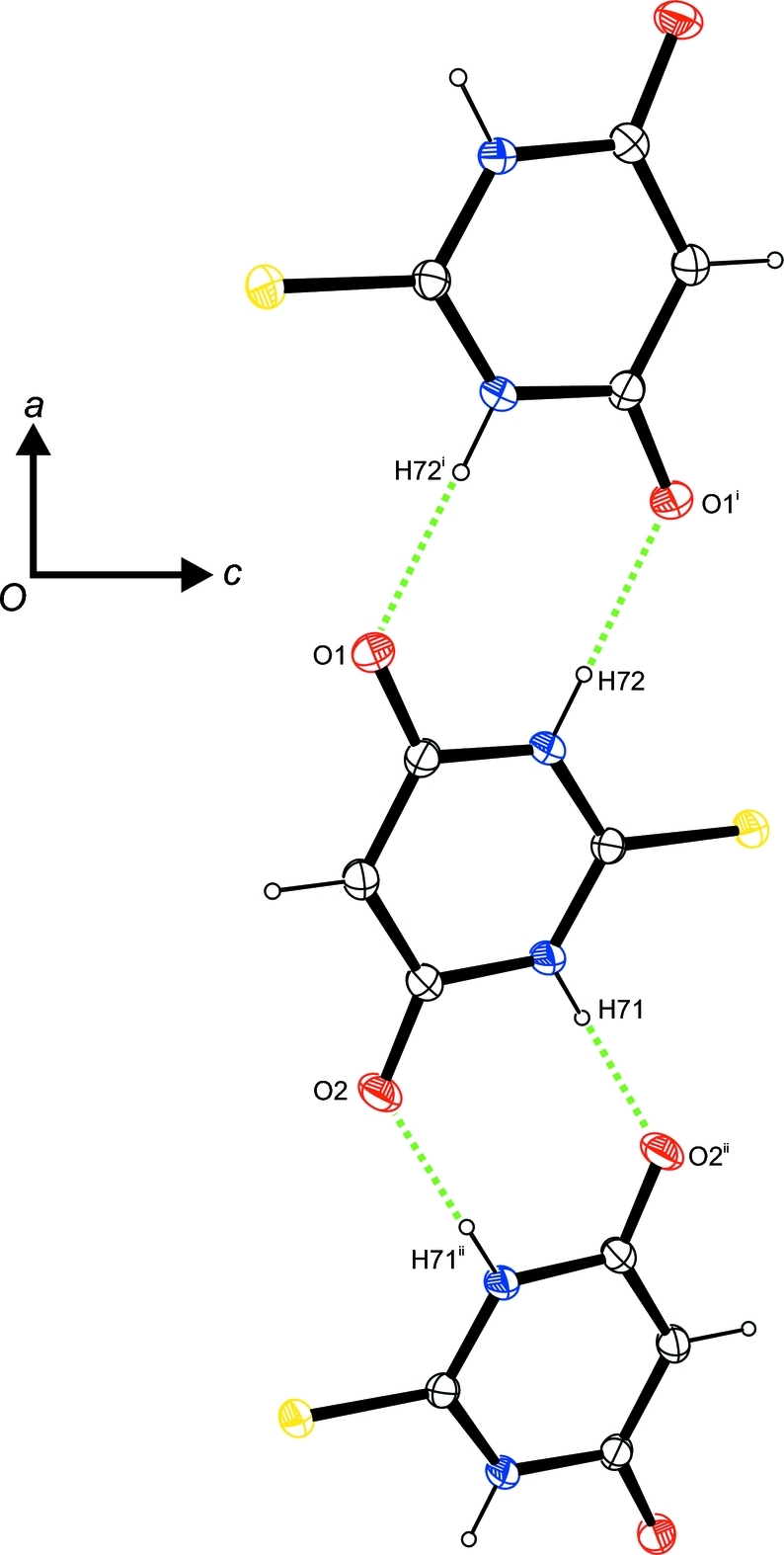
Intermolecular contacts among the thiobarbituric acid anions, viewed along [0 - 1 0]. Symmetry operators: (i) -x, -y + 1, -z + 1; (ii) -x + 1, -y + 2, -z + 1.

**Fig. 3.**
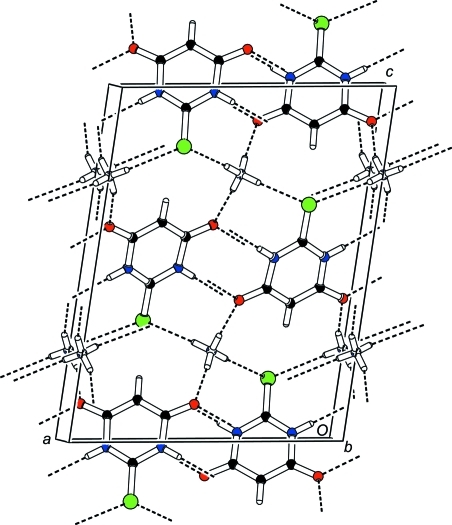
Hydrogen bonding system in the crystal structure of the title compound, viewed along [0 - 1 0]. Colour code for the atoms: green – sulfur, red – oxygen, blue – nitrogen, black – carbon; hydrogen atoms are shown as end of sticks.

**Fig. 4.**
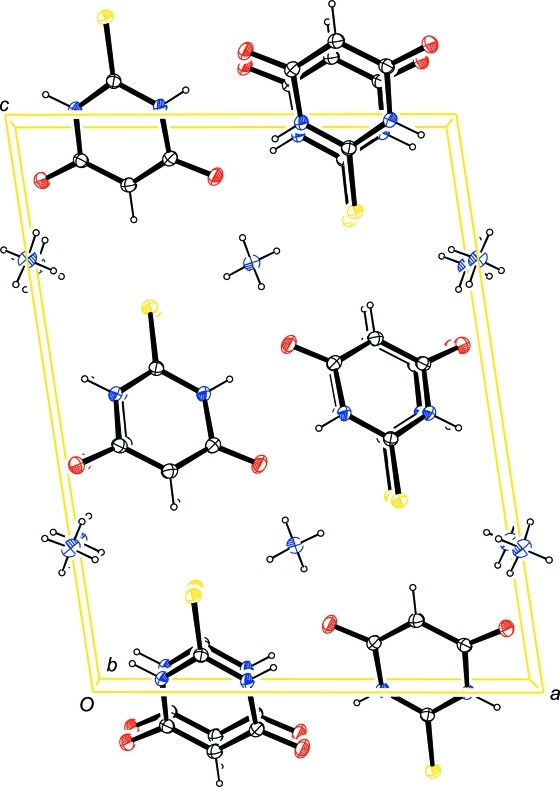
Molecular packing of the title compound, viewed along [0 1 0].

## supplementary crystallographic information

### Comment

Multidentate ligands play a major role in the synthesis of coordination polymers and metal-organic framework compounds (MOFs). Especially derivatives of benzoic acid have found widespread use in this aspect and a variety of these coordination polymers have been characterized in solution and in the solid state. Owing to the desire to synthesize functionalized MOFs whose poresizes or even complete architectural set-ups might easily be influenced upon variation of external parameters such as pH value or the presence and concentration of molecules that might reside inside the pores of these MOFs, chelating ligands related to benzoic acid but with the ability to change their bonding behaviour are necessary. In this aspect, 2-thiobarbituric acid seemed of interest since it may adopt several tautomeric forms whose persistence can be influenced by such external parameters. In order to gather structural information to allow for the tailored synthesis of MOFs based on thiobarbituric acid, we determined the crystal structure of its ammonium salt. The crystal structures of 2-thiobarbituric acid, its hydrate as well as several of its tautomeric forms are apparent in the literature (Calas & Martinez (1967); Chierotti *et al.* (2010)).

The thiobarbituric acid anion is present in its diketonic tautomeric form according to C—O bond lengths. Deprotonation took place on the methylene group. The intracyclic angles span a range from 116.64 (9)–124.67 (9) ° with the biggest angles invariably found on the nitrogen atoms and the smallest angle present on the sulfur-bonded carbon atom. The unit cell comprises two half-occupied ammonium cations (Fig. 1). The small puckering amplitude of the six-membered ring precludes a conformation analysis.

The crystal structure is dominated by hydrogen bonds. All of the ammonium cations' hydrogen atoms act as donors while both ketonic oxygen atoms of the heterocycle as well as the sulfur atom act as acceptors. The intracyclic NH groups participate in hydrogen bonds as well, however, they only have the ketonic oxygen atoms as acceptors. The latter intermolecular interactions connect the thiobarbituric acid anions to chains along [1 1 0] where each dimeric subunit shows inversion symmetry (Fig. 2). In terms of graph-set analysis (Etter *et al.* (1990); Bernstein *et al.* (1995)), the descriptor for the hydrogen bonds giving rise to these chains is *R*^2^_2_(8)*R*^2^_2_(8) on the unitary level. Taking into account the van-der-Waals radii of the atoms present in the crystal structure, the carbon-bond H atom participates in a C–H···S contact, which is, however, not very pronounced. In total, the molecules in the unit cell are connected to a three-dimensional network where layers of thiobarbituric acid anions are orientated parallel as well as approximately perpendicular towards each other (Fig. 3).

The packing of the compound in the crystal is shown in Figure 4.

### Experimental

2-Thiobarbituric acid was obtained commercially (Aldrich). Upon dissolution in hot, aqueous ammonia (*c* = 1.0 *M*) and subsequent cooling to room temperature, crystals suitable for the X-ray diffraction study were obtained.

### Refinement

The carbon-bound H-atom was placed in a calculated position (C—H 0.95 Å) and was included in the refinement in the riding model approximation, with *U*(H) set to 1.2*U*_eq_(C). All other H atoms were located on a difference map and refined as riding on their parent atoms with individual isotropic displacement parameters.

### Crystal data

**Table:**

NH_4_^+^·C_4_H_3_N_2_O_2_S^−^	*F*(000) = 336
*M**_r_* = 161.19	*D*_x_ = 1.651 Mg m^−^^3^
Monoclinic, *P*2/*c*	Mo *K*α radiation, λ = 0.71073 Å
Hall symbol: -P 2yc	Cell parameters from 1604 reflections
*a* = 11.3308 (4) Å	θ = 3.1–28.3°
*b* = 3.9396 (1) Å	µ = 0.44 mm^−^^1^
*c* = 14.6945 (5) Å	*T* = 200 K
β = 98.644 (1)°	Rod, colourless
*V* = 648.49 (4) Å^3^	0.53 × 0.19 × 0.08 mm
*Z* = 4	

### Data collection

**Table:**

Bruker APEXII CCD diffractometer	1604 independent reflections
Radiation source: fine-focus sealed tube	1476 reflections with *I* > 2σ(*I*)
graphite	*R*_int_ = 0.017
φ and ω scans	θ_max_ = 28.3°, θ_min_ = 1.8°
Absorption correction: multi-scan (*SADABS*; Bruker, 2008)	*h* = −14→15
*T*_min_ = 0.876, *T*_max_ = 1.000	*k* = −4→5
6071 measured reflections	*l* = −16→19

### Refinement

**Table:**

Refinement on *F*^2^	Primary atom site location: structure-invariant direct methods
Least-squares matrix: full	Secondary atom site location: difference Fourier map
*R*[*F*^2^ > 2σ(*F*^2^)] = 0.023	Hydrogen site location: inferred from neighbouring sites
*wR*(*F*^2^) = 0.067	H atoms treated by a mixture of independent and constrained refinement
*S* = 1.10	*w* = 1/[σ^2^(*F*_o_^2^) + (0.0357*P*)^2^ + 0.2247*P*] where *P* = (*F*_o_^2^ + 2*F*_c_^2^)/3
1604 reflections	(Δ/σ)_max_ = 0.001
116 parameters	Δρ_max_ = 0.33 e Å^−^^3^
0 restraints	Δρ_min_ = −0.20 e Å^−^^3^

### Fractional atomic coordinates and isotropic or equivalent isotropic displacement parameters (Å^2^)

**Table:**

	*x*	*y*	*z*	*U*_iso_*/*U*_eq_	
S1	0.25562 (2)	1.03184 (7)	0.667084 (17)	0.01875 (10)	
O1	0.05012 (7)	0.4191 (2)	0.39646 (5)	0.02247 (19)	
O2	0.44635 (7)	0.7956 (3)	0.39645 (6)	0.0262 (2)	
N1	0.34683 (8)	0.8796 (3)	0.51685 (6)	0.01740 (19)	
H71	0.4077 (15)	0.978 (4)	0.5427 (12)	0.027 (4)*	
N2	0.15417 (8)	0.7040 (2)	0.51663 (6)	0.01649 (19)	
H72	0.0908 (14)	0.678 (4)	0.5433 (10)	0.024 (4)*	
C1	0.25140 (9)	0.8614 (3)	0.56115 (7)	0.0151 (2)	
C2	0.14770 (9)	0.5599 (3)	0.42919 (7)	0.0160 (2)	
C3	0.24801 (9)	0.5883 (3)	0.38550 (7)	0.0182 (2)	
H3	0.2466	0.4960	0.3256	0.022*	
C4	0.35073 (9)	0.7513 (3)	0.42884 (7)	0.0177 (2)	
N90	0.5000	0.4019 (4)	0.2500	0.0202 (3)	
H901	0.5638 (14)	0.272 (4)	0.2710 (11)	0.034 (4)*	
H902	0.4821 (15)	0.527 (4)	0.2944 (11)	0.031 (4)*	
N91	0.0000	0.9763 (4)	0.2500	0.0234 (3)	
H911	0.0284 (17)	1.109 (5)	0.2961 (13)	0.048 (5)*	
H912	0.0539 (19)	0.853 (6)	0.2290 (15)	0.064 (6)*	

### Atomic displacement parameters (Å^2^)

**Table:**

	*U*^11^	*U*^22^	*U*^33^	*U*^12^	*U*^13^	*U*^23^
S1	0.01756 (15)	0.02527 (16)	0.01378 (14)	0.00002 (10)	0.00348 (10)	−0.00417 (9)
O1	0.0173 (4)	0.0325 (4)	0.0175 (4)	−0.0085 (3)	0.0023 (3)	−0.0039 (3)
O2	0.0176 (4)	0.0417 (5)	0.0209 (4)	−0.0090 (4)	0.0085 (3)	−0.0104 (4)
N1	0.0138 (4)	0.0247 (5)	0.0139 (4)	−0.0039 (4)	0.0025 (3)	−0.0034 (4)
N2	0.0136 (4)	0.0234 (5)	0.0130 (4)	−0.0027 (3)	0.0037 (3)	−0.0001 (3)
C1	0.0153 (4)	0.0169 (5)	0.0131 (4)	0.0005 (4)	0.0020 (4)	0.0017 (4)
C2	0.0159 (4)	0.0190 (5)	0.0127 (5)	−0.0013 (4)	0.0007 (4)	0.0012 (4)
C3	0.0184 (5)	0.0237 (5)	0.0128 (5)	−0.0030 (4)	0.0031 (4)	−0.0022 (4)
C4	0.0167 (5)	0.0228 (5)	0.0141 (4)	−0.0014 (4)	0.0043 (4)	−0.0012 (4)
N90	0.0193 (6)	0.0243 (7)	0.0170 (6)	0.000	0.0032 (5)	0.000
N91	0.0251 (7)	0.0260 (7)	0.0204 (7)	0.000	0.0074 (6)	0.000

### Geometric parameters (Å, °)

**Table:**

S1—C1	1.6893 (10)	N2—H72	0.874 (16)
O1—C2	1.2656 (13)	C2—C3	1.3914 (15)
O2—C4	1.2592 (13)	C3—C4	1.3963 (14)
N1—C1	1.3452 (14)	C3—H3	0.9500
N1—C4	1.3955 (13)	N90—H901	0.902 (17)
N1—H71	0.832 (17)	N90—H902	0.866 (17)
N2—C1	1.3453 (13)	N91—H911	0.88 (2)
N2—C2	1.3965 (13)	N91—H912	0.87 (2)
			
C1—N1—C4	124.67 (9)	O1—C2—N2	116.73 (10)
C1—N1—H71	118.4 (12)	C3—C2—N2	117.27 (9)
C4—N1—H71	116.9 (12)	C2—C3—C4	120.62 (10)
C1—N2—C2	124.11 (9)	C2—C3—H3	119.7
C1—N2—H72	120.3 (10)	C4—C3—H3	119.7
C2—N2—H72	115.5 (10)	O2—C4—N1	116.68 (9)
N1—C1—N2	116.64 (9)	O2—C4—C3	126.64 (10)
N1—C1—S1	120.74 (8)	N1—C4—C3	116.68 (9)
N2—C1—S1	122.62 (8)	H901—N90—H902	109.4 (14)
O1—C2—C3	125.99 (10)	H911—N91—H912	114.2 (19)
			
C4—N1—C1—N2	0.76 (16)	O1—C2—C3—C4	179.77 (11)
C4—N1—C1—S1	−178.90 (8)	N2—C2—C3—C4	0.82 (16)
C2—N2—C1—N1	0.48 (16)	C1—N1—C4—O2	178.58 (11)
C2—N2—C1—S1	−179.87 (8)	C1—N1—C4—C3	−1.12 (16)
C1—N2—C2—O1	179.70 (10)	C2—C3—C4—O2	−179.40 (12)
C1—N2—C2—C3	−1.25 (16)	C2—C3—C4—N1	0.27 (16)

### Hydrogen-bond geometry (Å, °)

**Table:**

*D*—H···*A*	*D*—H	H···*A*	*D*···*A*	*D*—H···*A*
N1—H71···O2^i^	0.832 (17)	1.971 (17)	2.8025 (12)	179.0 (16)
N2—H72···O1^ii^	0.874 (16)	1.976 (16)	2.8483 (12)	175.7 (15)
N90—H901···S1^iii^	0.902 (17)	2.426 (16)	3.3252 (8)	174.8 (14)
N90—H902···O2	0.866 (17)	1.927 (17)	2.7929 (12)	177.7 (16)
N91—H911···O1^iv^	0.88 (2)	1.90 (2)	2.7622 (13)	166.0 (18)
N91—H912···O1^v^	0.87 (2)	2.66 (2)	3.0669 (15)	110.0 (16)
N91—H912···S1^vi^	0.87 (2)	2.62 (2)	3.3069 (3)	136.0 (19)

Symmetry codes: (i) −*x*+1, −*y*+2, −*z*+1; (ii) −*x*, −*y*+1, −*z*+1; (iii) −*x*+1, −*y*+1, −*z*+1; (iv) *x*, *y*+1, *z*; (v) −*x*, *y*, −*z*+1/2; (vi) *x*, −*y*+2, *z*−1/2.
